# Toward the Real-Time and Rapid Quantification of Bacterial Cells Utilizing a Quartz Tuning Fork Sensor

**DOI:** 10.3390/mi14061114

**Published:** 2023-05-25

**Authors:** Abeer Alshammari, Sabaa T. Abdulmawla, Reem Alsaigh, Khaloud Mohammed Alarjani, Norah Salim Aldosari, Muthumareeswaran Muthuramamoorthy, Abdulaziz K. Assaifan, Hamad Albrithen, Khalid E. Alzahrani, Abdullah N. Alodhayb

**Affiliations:** 1 Department of Physics and Astronomy, College of Science, King Saud University, Riyadh 11451, Saudi Arabia; 2 Department of Botany and Microbiology, College of Science, King Saud University, Riyadh 11451, Saudi Arabia; 3 Department of Biomedical Technology, College of Applied Medical Sciences, King Saud University, Riyadh 11451, Saudi Arabia

**Keywords:** quartz tuning forks, quantification, bacteria, MEMS sensors

## Abstract

The quantitative evaluation of bacterial populations is required in many studies, particularly in the field of microbiology. The current techniques can be time-consuming and require a large volume of samples and trained laboratory personnel. In this regard, on-site, easy-to-use, and direct detection techniques are desirable. In this study, a quartz tuning fork (QTF) was investigated for the real-time detection of *E. coli* in different media, as well as the ability to determine the bacterial state and correlate the QTF parameters to the bacterial concentration. QTFs that are commercially available can also be used as sensitive sensors of viscosity and density by determining the QTFs’ damping and resonance frequency. As a result, the influence of viscous biofilm adhered to its surface should be detectable. First, the response of a QTF to different media without *E. coli* was investigated, and Luria–Bertani broth (LB) growth medium caused the largest change in frequency. Then, the QTF was tested against different concentrations of *E. coli* (i.e., 10^2^–10^5^ colony-forming units per milliliter (CFU/mL)). As the *E. coli* concentration increased, the frequency decreased from 32.836 to 32.242 kHz. Similarly, the quality factor decreased with the increasing *E. coli* concentration. With a coefficient (*R*) of 0.955, a linear correlation between the QTF parameters and bacterial concentration was established with a 26 CFU/mL detection limit. Furthermore, a considerable change in frequency was observed against live and dead cells in different media. These observations demonstrate the ability of QTFs to distinguish between different bacterial states. QTFs allow real-time, rapid, low-cost, and non-destructive microbial enumeration testing that requires only a small volume of liquid sample.

## 1. Introduction

Most studies, especially microbiology studies, necessitate the quantitative evaluation of bacterial populations. To assess food products’ safety, it may be possible to utilize the information collected, for example, to detect any foodborne pathogens [1]. In addition, information about contaminant counts could be used to study the efficacy of antimicrobial agents and processes [2].

Some live bacteria can exist in two separate states: the viable and culturable state (VC), which is capable of being cultured on proper media and developing into colonies, or the viable but non-culturable (VBNC) state [3]. VBNC cells are live cells that have lost their ability to divide or grow on standard media. Any bacteria may enter the VBNC state because of a number of factors, including antibiotic pressure, high or low temperatures, starvation, chlorination, pH changes, and oxygen stress [4]. The viable but unculturable stage is reversible because the cells retain their cellular activities and thus may regain their ability to reproduce, posing a health concern [5]. If VBNC cells are present, the colony-forming unit (CFU) count method will underestimate the total number of viable bacteria in a sample because VBNC cells are non-culturable. More worrisome than this, the non-detection of bacteria can lead to samples being regarded as free of pathogens. Bacterial species that cause human infections may pose serious risks to the public if viable cells are underestimated or not detected in quality control samples from food and water distribution systems or from clinical samples. It is highly desirable to be able to detect different states of bacteria using an accurate and fast method. However, even though a wide range of monitoring techniques are available, there is an ongoing need to develop new technologies that can monitor lower concentrations of bacteria, single cells, and other physical characteristics.

Quantitative techniques, such as direct plate counting [6], are used in order to determine the number of bacteria present in samples. In this case, the plates must be prepared by the serial dilution of samples to ensure the growth of single-cell colonies and are then incubated for a pre-specified amount of time (typically overnight or longer) for the colonies to become visible. However, long analysis times for slow-growing microbial strains can delay the initiation of appropriate antimicrobial medical therapy in clinical applications. In addition, long analysis times entail excessive costs in the manufacturing of pharmaceutical and medical products. Culture-independent approaches have been developed to circumvent these constraints, such as staining bacteria with fluorescence dyes, which is the most popular approach for determining the presence of VBNC bacteria [7,8] and for spectrophotometric (turbidimetric) analysis [9]. These techniques, nevertheless, may sometimes end in significant increases in costs due to the need for skilled personnel, pricey tools or consumables, or an increased limit for detection.

Mechanical biosensors are well known for being effective analytical tools because of their inexpensive manufacturing, quick analysis, and quick response. Currently, exploration is underway to see how quartz tuning forks (QTFs) can be used as biosensors to reduce the above-mentioned challenges. For the quick, accurate, and label-free detection of *E. coli*, a quartz crystal microbalance nanoplatform has been employed, and the frequency of the quartz crystal microbalance biosensor has been shown to be directly proportional to the antigen concentration under ideal circumstances [10]. In situ biofilm formation has also been detected using vibrating piezoelectric elements in a quartz crystal microbalance (QCM), as the viscoelastic characteristics of the attached bacteria alter the mechanical properties of the resonator [11,12]. Meanwhile, commercially available QTFs have been used as sensitive sensors of viscosity and density by measuring the damping and resonance frequency of the QTFs [13,14], and it has been shown to be possible to determine the influence of viscous biofilm attached to the surface of QTFs by measuring the vibration ringdown of said QTFs placed in a high-damping environment [15]. QTFs are constructed from two metal prongs attached at one end, which resonate at a specific frequency. This frequency varies according to the tuning fork’s shape, size, and material [16]. Since tuning forks utilize two tines to function, dampened losses are minimized. Since quartz crystal is inherently stiff, a quartz tuning fork retains the prong’s acoustic energy. This enables high-quality factors in the tens of thousands to be achieved in a vacuum at a fundamental frequency of 32,768 Hz [17]. The use of quartz then enables piezoelectric detection of tine motion, allowing for the construction of simple measuring arrays.

The fork behaves as a harmonic oscillator, and its performance is primarily determined by the resonance frequency, *f*, which is determined by the spring constant, *k*, and the effective mass, *m*, as well as the quality factor, *Q*, which is dependent on the surrounding medium. The formula governs the operation of QTFs as sensors, which is based on a change in resonance frequency, given by:

(1) $$f=\frac{1}{2\pi }\sqrt{\frac{k+\Delta k}{m+\Delta m}}$$

where *k* and *m* refer to the QTF’s force constant and mass, and *k* + ∆*k* and *m* + ∆*m* refer to the effective force constant and mass after loading, respectively [18].

The stability of the resulting frequency around the resonance is determined by the quality factor, which is defined as the ratio of the resonator’s stored energy to the energy dissipated during each period. The fundamental definition of *Q* is this ratio.

Small changes in these parameters can cause the resonance frequency to shift to a higher or lower value depending on which parameter is dominant. Due to mass loading, the resonance frequency changes to a lower value, whereas increasing stiffness causes the resonance frequency to shift to a higher value [17]. This property has the potential to be used as a sensor. Furthermore, fluid density and viscosity influence the resonance frequency and quality factor of the QTF sensor. It is commonly believed that the tuning fork sensor’s resonance frequency decreases with increasing liquid density and that its quality factor decreases with increasing liquid viscosity [19].

The objective of this study was to validate our hypothesis that a QTF sensor can detect the presence of bacteria in a liquid medium and can determine whether the bacteria is VC, VBNC, or dead. Another aim was to relate the QTF’s resonance frequency to the concentration of bacteria in the solution. On the other hand, optical density (OD) measurements at a wavelength of 600 nm are the most common method for estimating the number of cells in a solution because they are fast, inexpensive, simple, and relatively non-disruptive. However, turbidity measurements were found to be ineffective for counting bacterial concentrations below 10^5^ (CFU/mL). In this study, we focused on detecting very low concentrations of *E. coli* ranging between 10^2^ and 10^5^ CFU/mL. The principle behind the sensor is that a change in the environment surrounding its surface and an increase in mass loading brought on by the adsorption of biomolecules on the tuning fork’s surface causes a change in the measured frequency response and quality factor. The QTF system has a great deal of potential for overcoming some of the drawbacks of conventional approaches because of its high sensitivity, quick response, and high throughput capability.

## 2. Materials and Methods

Quartz tuning forks were removed from hermetic casings and mounted on a piezoelectric plate. Then, their output frequency was measured and recorded; according to a frequency resolution of 0.01 or 0.001 Hz, the measuring time was set to either 1 or 10 s. Next, a thin metal wire on one of the tines was glued to an electrode so that the piezoelectric sensory signal could be obtained. We employed a quartz crystal microbalance sensor device that was excited by a laboratory crystal oscillator. The output frequency was monitored, and the measurement time was set to 1 s. When the piezoelectric plate was activated, the fundamental frequency of resonance occurred, recorded by an amplifier locking in. The tuning fork’s amplitude and phase were calculated and stored on a computer for later analysis.

### 2.1. Bacterial Culture

*E. coli* strains were used to test the growth of the bacterial strains in this study. These strains were obtained from departmental stocks (Department of Botany and Microbiology, King Saud University)*. E. coli* cells were cultured aerobically at 37 °C in Luria–Bertani broth medium (LB; Oxoid), which was sterilized by autoclaving (121 °C, 15 min, 15 psi) under shaking conditions (160 rpm) for 16 h. Bacterial cells were grown until they reached an OD_600_ of 0.5, which is an optical density equal to McFarland No. 0.5 (approximately 10^8^ CFU/mL). Then, they were diluted to the desired concentrations with LB or phosphate-buffered saline (PBS) for further use.

### 2.2. Method

The Quester Q10, a commercial instrument, was used to carry out the measurements (Fourien Inc., Edmonton, AB, Canada). The instrument and experimental setup were already published in [14]. The QTF was directly submerged in an 18 μL droplet that was placed on a glass slide in order to conduct the experiments with very low volume consumption. DI water, LB, PBS, and bacterial solution were all tested. Using a regular pipette, a droplet was placed on a glass slide for each experiment. The QTF was then positioned in relation to the droplet using the onboard microscope and a three-axis translation mechanism. The QTF was then gradually drawn closer to the droplet as the connected microscope’s live video feed was continuously watched on a computer. The position of the QTF’s prongs is critical. The QTF was placed so that there was minimal contact with the surface of the droplet. Failure to carry out this step could result in a short circuit effect. After 10 min, the measurements began to allow more mass loading. Every experiment was conducted at room temperature. Additionally, the QTF’s frequency was swept from 31,000 to 34,000 Hz using the software control of our system, with each cycle lasting no longer than 30 s. The impedance analyzer recorded the QTF’s response, and the sweep result was displayed on the computer screen.

## 3. Results

The purpose of this study was to investigate the QTF sensor’s ability to detect very low concentrations of a laboratory strain of *E. coli*. To study the sensitivity of the sensors, a QTF was used to detect 3 different concentrations of *E. coli* (10^2^, 10^3^, and 10^5^ CFU/mL). 

**Figure 1:**
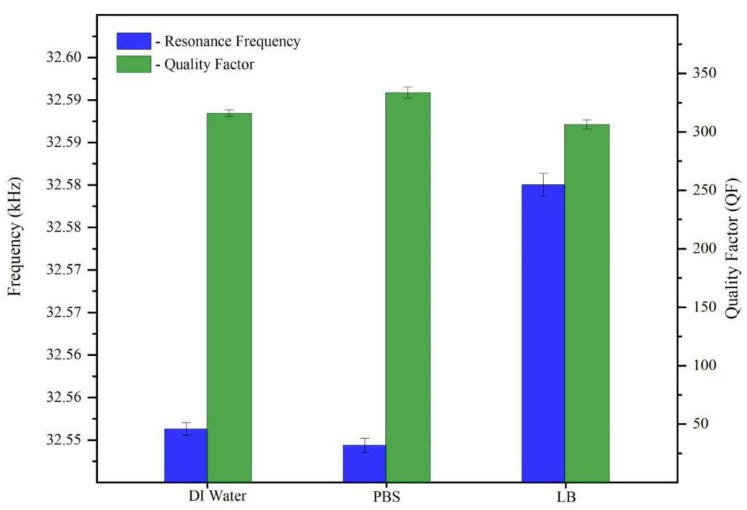
Comparison of changes in the resonance frequencies on the right (blue) and the quality factor (QF) on the left (green) of the QTF in DI water, LB, and PBS buffer.

First, the same QTF was subjected to DI water, bacterial growth medium (LB), and PBS buffer for comparison purposes. The resonance frequency of the QTF in DI water was found (in Figure 1) to be 32.550 kHz, which is very close to the resonance frequency in PBS, at 32.548 kHz, although the resonance frequency in LB was slightly higher at 32.580 kHz. According to Equation (1), the resonance frequency decreases with mass loading and increases with spring constant. In comparison to DI water, LB contains peptides, vitamins, and minerals, which causes its higher density, consequently leading to an increase in elasticity and a subsequent increase in the QTF’s resonance frequency in LB medium [17]. PBS buffer, conversely, contains only minerals and ions, which causes it to be less dense and viscous than LB and produces resonance frequencies that are comparable to those of DI water.

**Figure 2:**
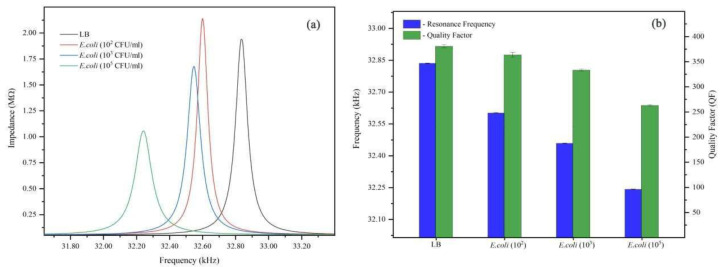
(**a**) The impedance versus frequency for different concentrations of bacteria, (**b**) comparison of changes in the resonance frequencies and *Q*-value of the QTF in different concentrations of *E. coli* bacteria.

To assess the ability of the QTF system to count bacteria levels in a solution, experiments were conducted under 3 different concentrations of *E. coli* bacteria in LB solution (10^2^, 10^3^, and 10^5^ CFU/mL). In this series of experiments, the same QTF was used to measure the response to LB alone, followed by *E. coli* solutions, with a cleaning step between each measurement. The QTF was cleaned by dipping it in 70% alcohol following each measurement. Figure 2a shows the impedance versus frequency for different concentrations of bacteria. Figure 2b, the resonance frequency decreased with the concentration, while the resonance peak broadened with the concentration due to the increased damping of the bacterial solution. At the lowest concentration of *E. coli*, 10^2^ CFU/mL, after 10 min of incubation, the resonance frequency significantly decreased to 32.600 kHz compared with its value in LB solution alone, which was 32.836 kHz. At higher concentrations (10^3^ and 10^5^ CFU/mL), the resonance frequency decreased with the concentration to 32.547 and 32.242 kHz, respectively, as can be seen in Figure 2b. Equation (1) shows that the resonance frequency of an oscillating QTF can only decrease if its mass increases or the spring constant *k* decreases. This implies that the interaction of *E. coli* with the QTF either reduces the overall stiffness of the QTF or increases its effective mass when compared with its value in LB solution alone. As a result, we conclude that the interaction of *E. coli* with QTF is such that the mass loading effect dominates at all tested concentrations of *E. coli*, reducing the resonance frequency of the QTF [18].

**Figure 3:**
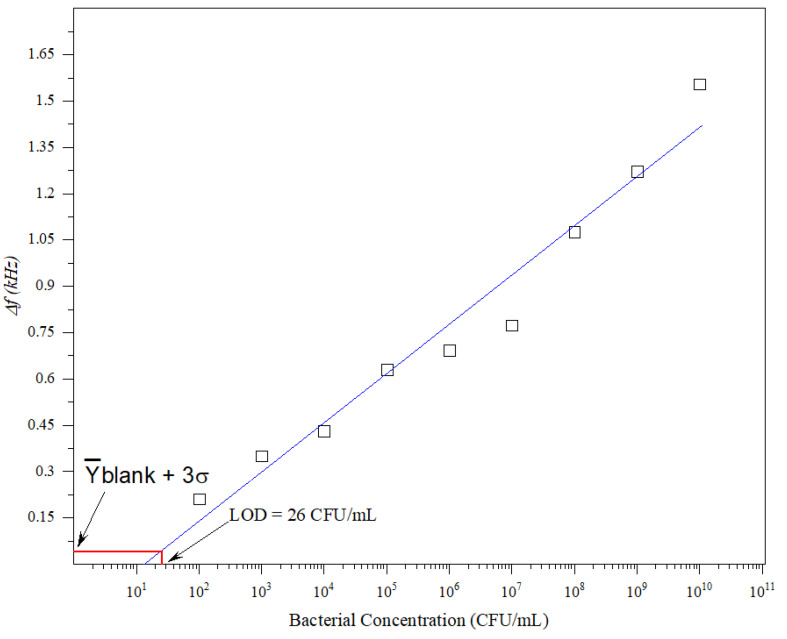
Calibration curve between the frequency shift and bacterial concentration.

The sensitivity of biosensors is denoted by the detection limit, which refers to the lowest concentration of analyte that can be detected reliably. A low detection limit is preferable because it allows infections to be detected at an early stage. The calibration curve was obtained by plotting different *E. coli* concentrations (10^2^–10^10^ CFU/mL) against the frequency shift. Figure 3 shows the reported biosensor calibration curve. The LoD was obtained using Equation (2) [20,21].

(2) $$X_{LoD}=f^{-1}\left({\bar{y}}_{\mathrm{blank}}+3\sigma \right)$$

where $f^{-1}$ is the inverse function of the linear fitting shown in Figure 3. σ and $\bar{y}$_blank_ are the mean value of the standard deviation and readout of the blank (LB) samples, respectively. The LoD can be obtained by finding the intersection between the solid blue line and $\bar{y}$_blank_ + 3σ, as illustrated in Figure 3. According to Figure 3, the LoD is 26 CFU/mL.

The ability of a sensor to respond selectively to a particular target is referred to as selectivity. Because the surface of the QTF was uncoated, and because it is a mass-loading-based detection technique, any particle that interacts with the QTF’s surface will cause a frequency shift. As a result, the surface of the QTF must be functionalized with receptors capable of effectively capturing target bacteria or biomolecules onto the QTF’s surface. 

Van der Waals interactions, electrostatic interactions, steric force, and acid–base interactions are among the first physicochemical forces that bacteria encounter when moving from a liquid environment to a solid surface. It is well known that different mechanical restrictions apply to bacteria that swim near the surface compared with those that swim in a larger volume of liquid. Single cell tracking has shown that Brownian motion, which randomly modifies swimming in a bulk liquid environment, is amplified at the near-surface boundary, producing more circular trajectories near the surface but straighter trajectories farther away [22]. The probability of contact between the bacterium and the surface is increased because these random circular motions allow bacteria to spend more time near the surface than cells that swim in straight lines. Additionally, bacterial cells experience greater drag forces close to the surface, which results in a slower swimming velocity at the surface boundary than in a bulk liquid environment [22]. Because the flow rate in the drop can be negligible, we can expect a large boundary layer near the surface of a QTF.

**Figure 4:**
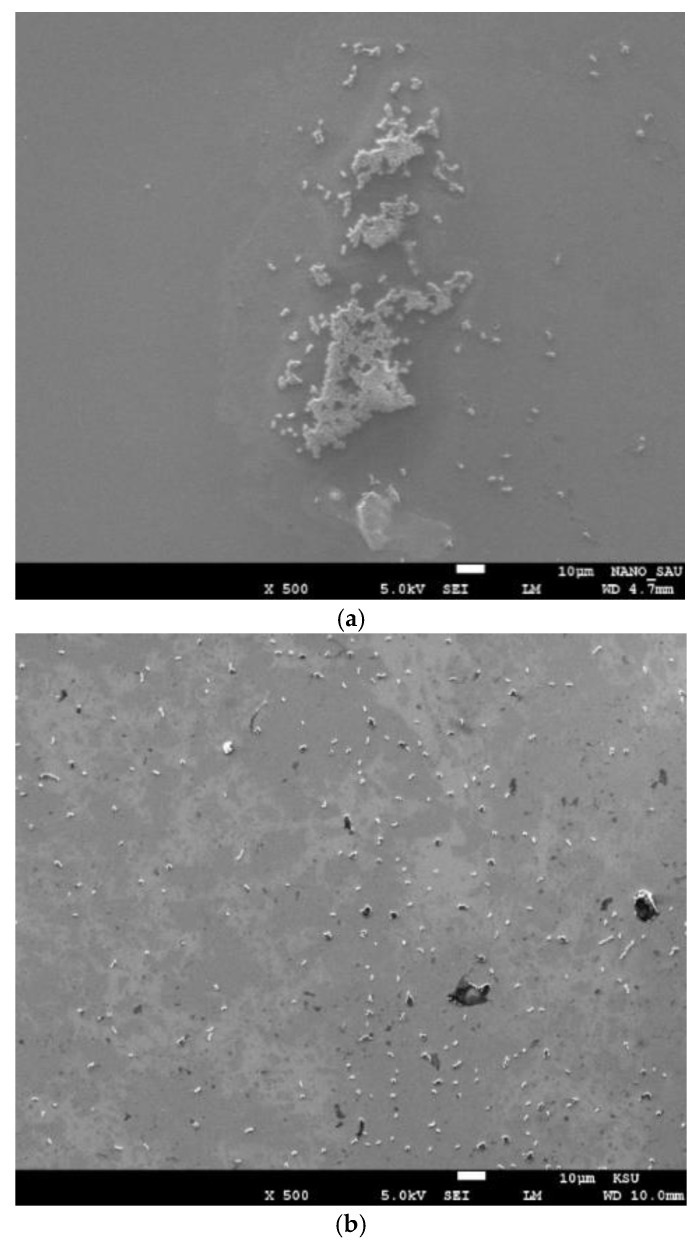
(**a**) SEM image of the incubated quartz surface washed once; (**b**) SEM image of the incubated quartz surface washed three times.

To determine whether the bacteria was attached to the surface irreversibly or reversibly, or simply in close proximity to it, the quartz surface was incubated with a 10^3^ CFU/mL concentration of *E. coli* for 10 min, then washed 1 or 3 times to remove non-attached cells, followed by imaging using SEM, as can be seen in Figure 4. The images confirm the weak attachment of the bacteria to the quartz surface, which was easily removed by washing. It has been discovered that *E. coli* cells attached to stiff substrates move faster than those attached to soft substrates [23], which suggests that detachment rates from stiff surfaces may be higher [23]. Based on this, we believe that physical forces—probably Van der Waals and steric forces—cause bacteria to adhere reversibly onto the surface of the QTF, and that the vibration of the prongs also contributes to the intermittent nature of bacteria attachment–detachment on the QTF’s prongs during the timescale of the experiment.

From another perspective, close to the surface of the QTF, organic molecules in the bulk liquid or those secreted by bacteria are capable of being adsorbed onto the surface to form a conditioning film. A variety of (macro) molecules, such as proteins, amino acids, lipids, and polysaccharides, may be present in such films. The resonance frequency of the QTF is affected by both the density and viscosity of the medium surrounding its two prongs and the conditioning film on its surface. When bacteria bind to a surface, energy is lost due to the viscosity of a liquid medium or the cells’ ability to trap water, which dampens the oscillation of the QTF [24]. The energy dissipation in the vibrating prongs is proportional to the resonance peak broadening, and the greater the broadening, the greater the losses [14]. The quality factor *Q* can be calculated as the ratio of the resonance frequency to the resonance curve’s Full Width at Half Maximum (FWHM) value ∆*f* [16].

(3) $$Q=\frac{f_{o}}{\Delta f}$$

We extracted the corresponding quality factors for our QTF from the resonance width and resonance frequency of the tuning fork’s response in DI water, LB, and PBS (Figure 1) and different concentrations of *E. coli* (Figure 2b). 

DI water had a *Q*-value of 314, which reduced to 303 in the LB solution and increased to 330 in PBS (using the same fork in all 3 media, as in Figure 1). The viscosity of the LB medium was the highest, which is consistent with its quality value. The *Q*-factor values were found to be 360, 335, and 262 when the *E. coli* concentration was 10^2^, 10^3^, and 10^5^ CFU/mL, respectively, as can be seen in Figure 2b. The decreasing quality factor compared with the LB medium’s value for this fork, which was 378, indicates more energy dissipation. The energy dissipation is proportional to the rigidity of the QTF; a high dissipation value indicates the presence of a soft or viscous material in contact with the surface of the QTF, whereas a low dissipation value indicates the presence of a rigid material that follows the oscillation. Thus, we conclude that at low *E. coli* concentrations, the QTF’s resonance frequency was suppressed compared with LB, and a very thin layer of conditioning film was formed on the QTF’s prongs, resulting in the film’s ability to follow oscillations, leading to a slightly higher dissipation and a lower *Q*-value compared with LB alone. Moreover, the resonance frequency decreased, and a thicker layer of conditioning film formed at higher concentrations of *E. coli*, implying more energy dissipation and a lower *Q*-value. The combined frequency and dissipation data can reveal the rigidity and viscoelasticity of biomolecules adsorbed on the surface of the QTF.

**Figure 5:**
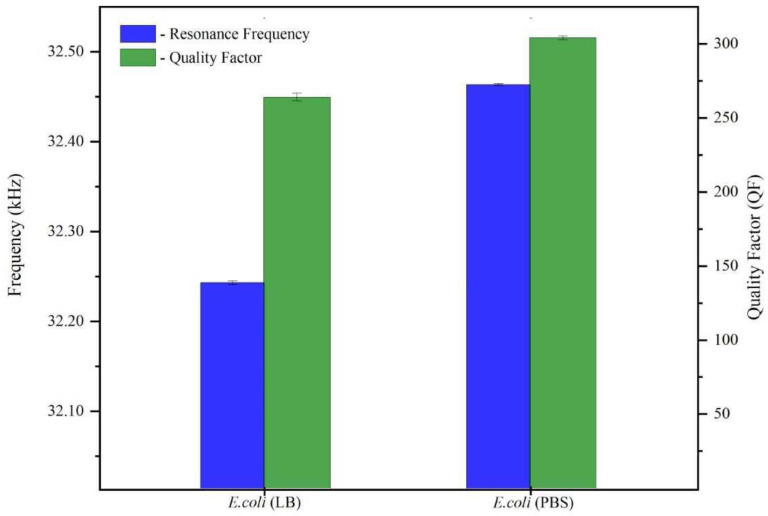
Resonance frequency and *Q*-factor for a 10^5^ CFU/mL concentration of *E. coli* in LB and PBS.

As an additional check of our system’s sensitivity to the presence of *E. coli* bacteria, the QTF was exposed to 10^5^ CFU/mL of bacteria in PBS medium. PBS is not a bacterial culture medium. It lacks the nutrients required for normal bacterial growth; instead, it provides a stable habitat in which germs can survive until they are transferred to more suitable conditions. The resonance frequency and *Q*-values for 10^5^ CFU/mL of *E. coli* in LB and PBS are depicted in Figure 5. Upon exposure to a bacterial solution in both media, the QTF’s resonance frequency and *Q*-factor decreased compared with the buffer media (LB and PBS), as shown in Figure 1. 

**Figure 6:**
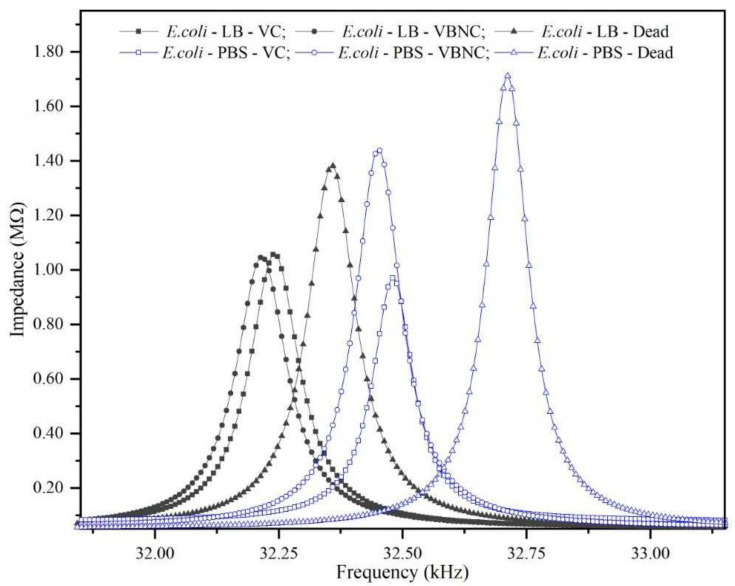
Comparison graph between dead and live cells (VC and VBNC) in LB and PBS media at a 10^5^ CFU/mL concentration.

Microbe viability is critical in determining the safety of food and drinking water and has significant consequences for environmental and medical microbiology [25]. To test our system’s capacity to detect cell viability, we incubated 2 samples of 10^5^ CFU/mL of *E. coli* in 2 different media (LB and PBS) at room temperature for 20 days to ensure that most cells were in a VBNC state due to nutrient depletion. Another sample of the same concentration was killed by autoclaving. Autoclaves use steam heat to raise temperatures to the point where proteins within a microbe’s cell walls break down and begin to coagulate, allowing cellular contents to leak into the surroundings. A comparison graph for living (VC and VBNC) cells and dead cells at the concentration of 10^5^ CFU/mL in LB and PBS is shown in Figure 6. In both media, the VBNC cells tended to shift the resonance frequency to slightly lower values than the VC cells, whereas dead cells tended to shift the resonance frequency to significantly higher values than the VC cells.

When a bacterial cell enters the VBNC phase, it undergoes numerous cellular changes, such as cell leakage and lower levels of metabolic activity [5]. Furthermore, the media have a lower nutrient density. Therefore, the QTF’s resonance frequency will shift slightly to lower values as a result of the decrease in media density. Conversely, it has been found that dead *E. coli* cells have a lower total mass than living *E. coli* cells [26]. Here, heat treatment caused bacteria to exist without rigid walls and modified the medium’s composition; consequently, the mass of bacteria decreased, as did their ability to adhere to the QTF’s prongs, resulting in an increase in resonance frequency values. The QTF could differentiate between live, VBNC, and dead cells. 

**Figure 7:**
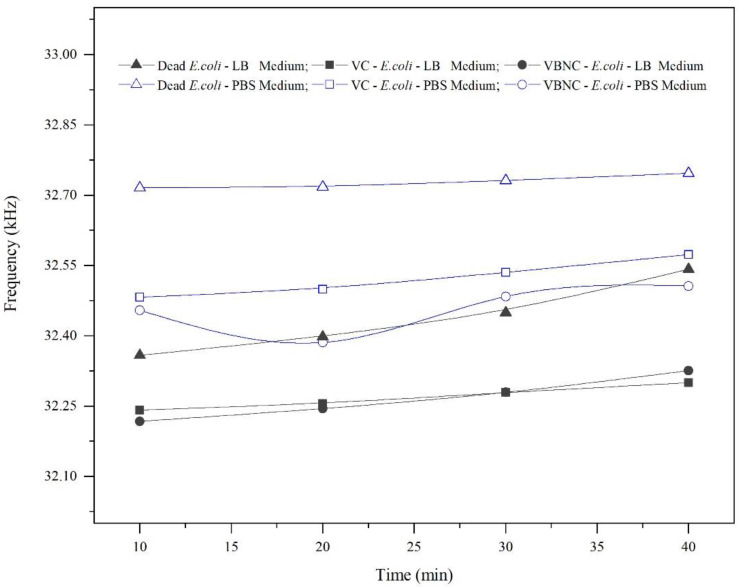
Comparison graph between dead and live cells (VC and VBNC) over time in LB and PBS media at a 10^5^ CFU/mL concentration.

The resonance frequency was measured over time (every 10 min until the drop began to evaporate and detach from the QTF’s prongs), as shown in Figure 7, keeping in mind that the QTF’s prongs were mounted so that they barely touched the surface of the droplet. Thus, after 40 min, although the laboratory temperature was kept under 21 °C, we noticed that the drop surface became lower and it continued to touch the prongs due to surface tension, implying that the area of the surface touching the prongs decreased. This shortcoming would limit its applicability for real-time monitoring of bacterial growth. In dead cells, the resonance frequency was nearly constant in PBS; however, it increased slightly in LB media by 80–90 Hz. This increase could be attributed to a decrease in the mass loading of dead cells over time, which have a lower ability to maintain attachment. Moreover, the resonance frequency increased insignificantly by approximately 50–60 Hz in VBNC and VC due to a decrease in mass loading. We suggest that VBNC and VC are more capable of maintaining attachment to the QTF’s surface, despite the weak attachment that was confirmed in Figure 4. Nevertheless, the values of the quality factor (not shown) were almost constant for all types of bacteria during the same period, which is likely due to the fact that the viscosity of the media was not changed significantly.

## 4. Conclusions

In this study, we investigated a QTF sensor with the goal of detecting the presence of bacteria in a liquid medium and determining the bacterial state (VC, VBNC, or dead). We found a correlation between the QTF’s response and the bacterial concentration in the solution. The QTF was tested against various concentrations of *E. coli* (ranging from 10^2^ to 10^5^ CFU/mL). Due to mass loading, as the *E. coli* concentration increased, the frequency decreased from 32.836 to 32.242 kHz. Similarly, the *Q*-factor values were 360, 335, and 262 when the concentrations of *E. coli* were 10^2^, 10^3^, and 10^5^ CFU/mL, respectively. Due to the formation of a conditioning film on the QTF’s prongs and an increase in the viscosity of the medium, the quality factor decreased as the concentration of *E. coli* increased, indicating greater energy dissipation and a lower *Q*-value. Upon exposure to a bacterial solution in a different medium (PBS), both the QTF’s resonance frequency and *Q*-factor decreased in comparison to buffer media. In order to determine cell viability, the QTF was exposed to 10^5^ CFU/mL of bacteria in LB and PBS media. In both media, VBNC cells shifted the resonance frequency to slightly lower values than VC cells, while dead cells shifted it to significantly higher values.

These findings show that the QTF system is capable of quantifying bacterial concentrations and that our system is highly sensitive to the presence of *E. coli* bacteria in any medium. However, selectivity of detection can be achieved by chemically modifying the surface of the QTF with biorecognition molecules such as antibodies or aptamers. The QTF was able to distinguish between live and dead cells, but more study and increased sensitivity are needed to distinguish between VC and VBNC cells. These results, when considered in their entirety, contribute to the ongoing challenge of developing technologies that can measure cell populations at low concentrations and measure the distinctive physical states of cells, both of which may be useful for the development of future diagnostics. To gain a deeper understanding of bacterial growth in relation to cell concentration and antimicrobial susceptibility testing, additional work is required to apply this strategy. One potential future study is to consider testing different types of bacteria (i.e., Gram positive and Gram negative) to further develop the QTF for biosensing applications.

## Data Availability

All data are presented in this study.
